# The Differences in the Developmental Stages of the Cardiomyocytes and Endothelial Cells in Human and Mouse Embryos at the Single-Cell Level

**DOI:** 10.3390/ijms25063240

**Published:** 2024-03-13

**Authors:** Chuyu Liu, Ning-Yi Shao

**Affiliations:** 1 Department of Biomedical Sciences, Faculty of Health Sciences, University of Macau, Taipa, Macau SAR 999078, China; yb97654@connect.um.edu.mo; 2 MoE Frontiers Science Center for Precision Oncology, University of Macau, Taipa, Macau SAR 999078, China; 3 Zhuhai UM Science and Technology Research Institute, Zhuhai 519000, China

**Keywords:** embryonic heart development, cardiomyocytes, human–mouse comparison, DTW, *Prdm16*, hPSCs

## Abstract

Our research focuses on expression patterns in human and mouse embryonic cardiomyocytes and endothelial cells at the single-cell level. We analyzed single-cell datasets containing different species, cardiac chambers, and cell types. We identified developmentally dynamic genes associated with different cellular lineages in the heart and explored their expression and possible roles during cardiac development. We used dynamic time warping, a method that aligns temporal sequences, to compare these developmental stages across two species. Our results indicated that atrial cardiomyocytes from E9.5 to E13.5 in mice corresponded to a human embryo age of approximately 5–6 weeks, whereas in ventricular cardiomyocytes, they corresponded to a human embryo age of 13–15 weeks. The endothelial cells in mouse hearts corresponded to 6–7-week-old human embryos. Next, we focused on expression changes in cardiac transcription factors over time in different species and chambers, and found that *Prdm16* might be related to interspecies cardiomyocyte differences. Moreover, we compared the developmental trajectories of cardiomyocytes differentiated from human pluripotent stem cells and embryonic cells. This analysis explored the relationship between their respective developments and provided compelling evidence supporting the relevance of our dynamic time-warping results. These significant findings contribute to a deeper understanding of cardiac development across different species.

## 1. Introduction

Cardiovascular disease remains a leading cause of mortality globally, driving the imperative for more effective preventive and therapeutic strategies. In this context, mouse models have become a cornerstone in cardiovascular research, providing invaluable insight into development and disease mechanisms [1,2]. Moreover, the differences in the cardiac anatomy and physiology of humans and mice have begun to be studied, using orthologous gene expression to understand the molecular changes behind the differences and the associated selective pressures [3,4]. The parallels and discrepancies between mice and human heart development demand careful exploration to optimize the translational outcomes [5,6,7,8,9].

Heart development in humans commences with cardiac progenitor cells around the 15th day of gestation, which corresponds to embryonic day 7.5 (E7.5) in mice [10,11]. This early initiation is consistent, while the pace of cardiac development varies markedly [12]. Mouse heart development progresses rapidly, with human-gestational-week-23 embryos being equivalent to mouse embryos at day 17.5 [5]. Despite these temporal differences, the anatomy of mouse and human hearts remains remarkably congruent throughout development, with the division of heart chambers following similar sequences [5,6,12,13]. The structure of the adult human heart predominantly comprises four cell types: cardiomyocytes (CMs), cardiac fibroblasts (FBs), mural cells (which include pericytes and smooth muscle cells), and endothelial cells (ECs) [14]. Prior research has indicated that cardiomyocytes occupy more than 70% of the volume of the mammalian heart [15]. Although the volume fraction was so high, the adult mouse cardiomyocytes numbered about 30% of the total volume, and 60% of the noncardiomyocytes were endothelial cells [16]. This also reveals the important role of this fraction of cells during heart development.

Comparative analyses of organ development across species that have included the heart have shed light on gene expression patterns and functions pertinent to organogenesis [3]. Further, the innovative use of the dynamic time-warping (DTW) algorithm in bulk RNA-seq has provided an effective approach for aligning the developmental time series of neural development across humans, chimpanzees, and macaques, revealing similarities and differences in organ development across different species [17]. Since then, at the bulk RNA-seq level, the DTW algorithm has found applications in aligning the developmental trajectories of various organs, including the heart, offering valuable perspectives on interspecies developmental patterns [4]. These studies have revealed conservative patterns of the transcriptome in mammals at different developmental stages. They have also provided an understanding of the comparable expression of the same organ across species.

The advent of single-cell RNA sequencing (scRNA-seq) has revolutionized our capacity to characterize transcriptionally distinct subpopulations throughout tissue development [18,19,20,21]. Previous studies have shown that, even in the heart, the same type of cell can exhibit different expression profiles in different anatomical structures [8,14,20]. One previous study that used scRNA-seq combined with the DTW algorithm enabled the pseudo-time alignment of the nervous system during organoid development across humans, chimpanzees, and macaques [22]. However, differences in the gene expression patterns during the development of specific types of cells in the developing heart are still elusive between species such as humans and mice.

Previous efforts have focused on aligning the developmental stages of mouse and human heart tissues using the heart’s developmental events (Carnegie stages) and bulk RNA sequencing [4,5,18]. However, the higher resolution provided by scRNA-seq allows for a more detailed understanding of these developmental stages. We leveraged the rich information offered by scRNA-seq datasets, specifically Cui’s dataset for humans, spanning the developmental stages from 5 weeks (5W) to 25 weeks (25W) of gestation [18], and Cao’s dataset for mice, covering embryonic day 9.5 (E9.5) to E13.5 [23]. Given the previous alignment results and morphological characteristics, it has been suggested that mouse gestational day E13.5 is within the 17th week of human gestation. We focused our analysis on the developmental stages from 5W to 17W in the human dataset [4,5,7,18]. By aligning the developmental stages of specific cardiac cell types between humans and mice, we found that, in the case of different chambers, even for the same cell type (cardiomyocytes), there were differences in the developmental stages between the species. The aim of this study was to elucidate the disparities in cardiac development between humans and mice at the single-cell level. By investigating these differences, we aimed to enhance our comprehension of the translational relevance and applicability of the mouse model in studying heart diseases, including congenital heart abnormalities and myocardial regeneration following a myocardial infarction.

## 2. Results

### 2.1. Identification of Cell Types in Human Embryonic Hearts

**Figure 1:**
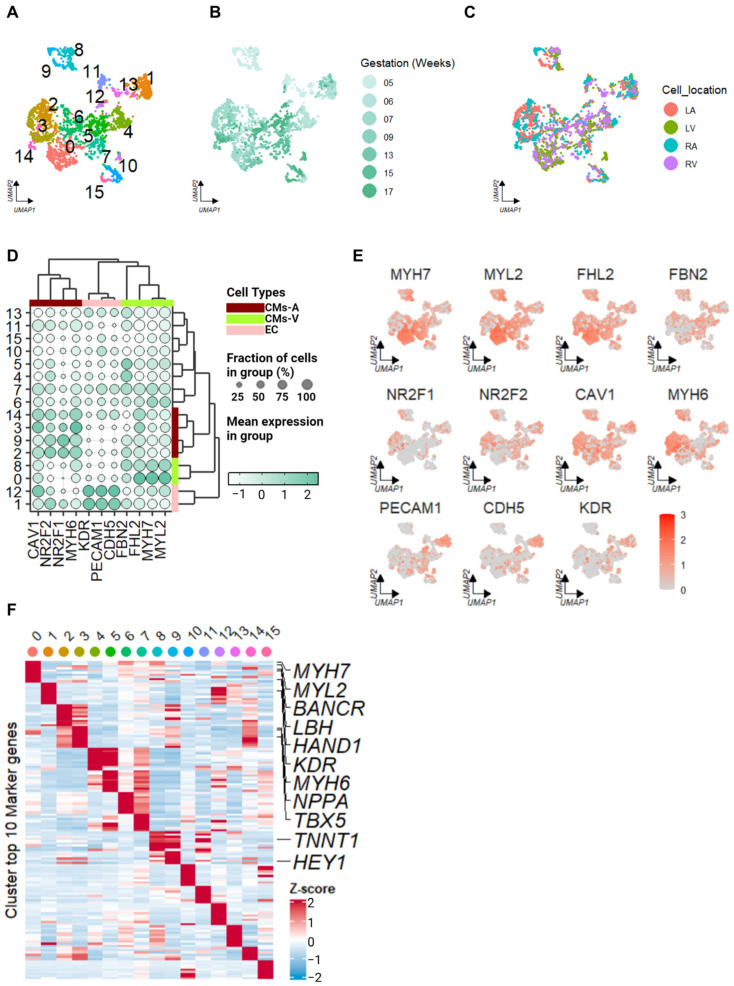
Cell types in the human embryonic heart, identified through an scRNA-seq analysis. The uniform manifold approximation and projection (UMAP) shows all filtered cells. (**A**) Unbiased clusters are indicated by different colors, identified using Louvain clustering. (**B**) The colors indicate the developmental stages from 5 weeks (5W) to 17W of gestation. (**C**) The colors represent the locations from which the cells were sourced. (**D**) Cell-type marker genes in the clusters were identified in (**A**). Bar: CMs-A, dark red; CMs-V, green; ECs, pink. The dot sizes represent the fraction of cells within each cluster. The color shades of the dots represent the mean expression level of the genes in the cluster. Same as below. (**E**) In the UMAP visualization, cardiomyocyte chamber-specific and endothelial cell-specific markers are highlighted. (**F**) The heatmap displays the Z-score-scaled average expression levels of DEGs for each cluster identified in (**A**) within the human embryonic heart.

By employing transcripts per million (TPM) values and converting them to log2 values (TPM + 1), we aimed to reduce the individual differences and batch effects in the single-cell datasets (this approach applies to all datasets within this study). The processed value was used as the input in the Seurat package. In this dataset (GSE106118), previous studies that employed the same approach have demonstrated minimal individual-specific differences. This approach has been mentioned in previous studies and has demonstrated that the individual differences are small [18]. After quality control, we obtained 2614 cardiac cells spanning the developmental stages from 5W to 17W of gestation. For a reduction in dimensionality (principal component analysis, PCA) and visualization, the top 18 principal components (PCs) were utilized for uniform manifold approximation and projection (UMAP). Consistent with previous findings, the 5W cardiomyocytes exhibited a distinct separation from the cells at other developmental stages (Figure 1A,B) [18]. Furthermore, we retrieved annotation information from the original research to investigate the atrial or ventricular origin of the cells (left atrium (LA), left ventricle (LV), right atrium (RA), and right ventricle (RV)) (Figure 1C).

Traditional chamber-specific markers are used in adult cardiomyocytes, such as *MYH7*, *MYL2*, and *FHL2* for the ventricles and *NPPA*, *MYL7*, and *MYL4* for the atria [14]; the latter might not be suitable for classifying embryonic cardiomyocytes [24]. During the embryonic stages, *MYL7* is expressed in both the atria and ventricles, and its expression gradually shifts to *MYL2* during the transition from fetal to adult subtypes [25]. To accurately identify the cell types in our analysis, we employed specific markers that have been identified in mouse embryo hearts [26]. For ventricular cardiomyocytes (CMs-V), we used the markers *MYH7*, *MYL2*, and *FHL2* [18]. For atrial cardiomyocytes (CMs-A), we used the markers *NR2F1*, *NR2F2*, and *CAV1* [26]. Similarly, for endothelial cells (ECs), we utilized the markers *PECAM1*, *KDR*, and *CDH5*, which were identified in a study of human fetal hearts [21]. We demonstrated the effectiveness of this series of markers in identifying chamber-specific cardiomyocytes and endothelial cells across species.

By applying Louvain clustering to the dataset, we annotated the clusters and determined the cell types based on the expression patterns of the aforementioned markers. This allowed us to classify the cardiomyocytes into CMs-A and CMs-V (Figure 1D). Notably, we observed the expression pattern of *MYH6*, a marker specific to CMs-A in human embryos, which clustered together with the markers we used for CMs-A identification. These findings are consistent with those of previous research [18]; CMs-V exhibited higher expression levels of extracellular matrix genes, such as *FBN2*, compared to CMs-A (Figure 1D,E). Based on the expression patterns of the marker genes, we designated C0 and C8 as CMs-V-like; C2, C3, C9, and C14 as CMs-A-like; and C1 and C12 as EC-like.

To further validate our cell type identification, we examined the correspondence between our obtained results and the cell anatomical labels in original research and found that they were similar (Figure 1C). To investigate differentially expressed genes (DEGs) in CMs-A, CMs-V, and ECs, we identified marker genes within each cluster and selected the top 10 genes with the largest fold change in each cluster (we performed the “FindAllMarkers” algorithm with the default parameters and confirmation of the adj. *p*-value for each marker < 0.01; this approach was applied to all single-cell DEG identifications within this study) (Figure 1F). The resulting heatmap provided insight into the enriched genes within each cell type. Notably, *BANCR* showed enrichment in 6- to 17-week-old CMs-V [27], in addition to commonly enriched markers such as *MYH7* and *MYL2*. Moreover, *TBX5*, *MYH6*, *NPPA*, *HEY1*, and *RELN* were highly enriched in CMs-A, while *HEY2*, *LBH*, and *HAND1* exhibited a higher expression in CMs-V [27].

### 2.2. Identification of Cell Types in the Mouse Embryonic Cardiac Muscle Lineages and Mouse Fetal Heart

**Figure 2:**
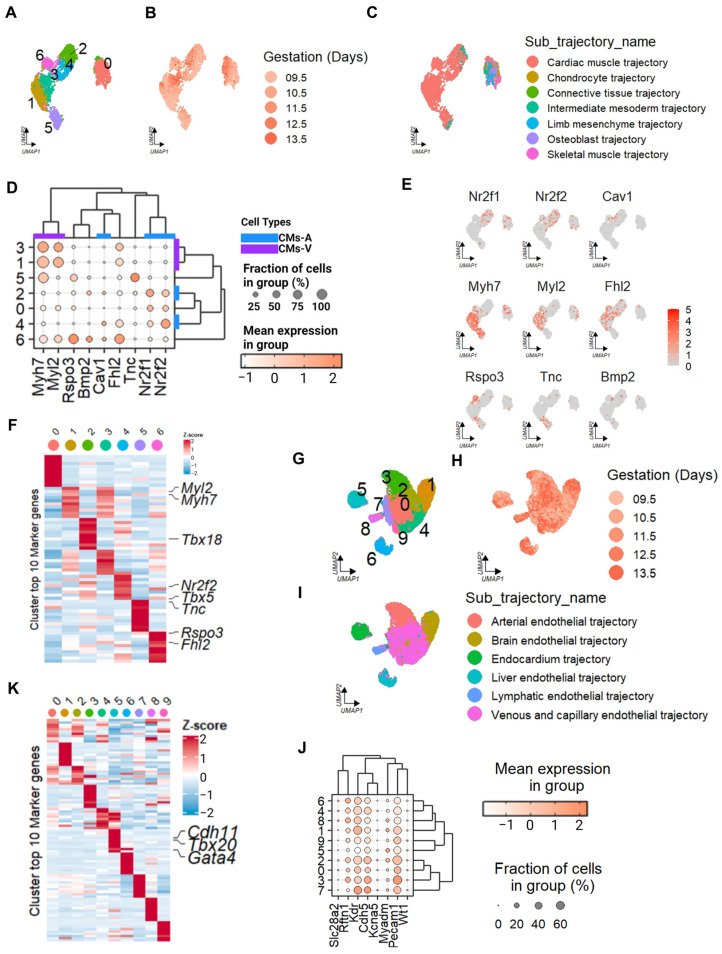
Cell types identified in the cardiac muscle and endothelial trajectory of mouse embryos through an scRNA-seq analysis. The UMAP visualization represents all cells within the cardiac muscle lineage. (**A**) The different colors represent distinct clusters. (**B**) The shades of color correspond to developmental stages, ranging from day 9.5 to day 13.5 of gestation. (**C**) The different colors show various trajectories. (**D**) The cell-type marker genes in the clusters identified in (**A**). Bar: CMs-A, blue; CMs-V, purple. (**E**) The UMAP visualization highlights the markers specific to cardiomyocyte chambers. (**F**) The heatmap indicates the Z-score-scaled average expression levels of DEGs for each cluster, as identified in (**A**), in the cardiac muscle. The color of the dot corresponds to those in (**A**). (**G**–**I**) All cells within the endothelial lineage are presented using UMAP visualization. (**G**) The different colors correspond to distinct clusters. (**H**) The shades of color represent developmental stages from day 9.5 to day 13.5 of gestation. (**I**) The various colors denote the trajectories of different cell types. (**J**) The cell-type marker genes in the clusters identified in (**G**). (**K**) The heatmap exhibits the Z-score-scaled average expression levels of DEGs within each cluster identified in G in the endothelial lineage. The color of the dot corresponds to those in (**G**).

We then utilized multiple mouse datasets to comprehensively investigate the characteristics of expression of cardiomyocytes within the whole embryo and the embryo heart (datasets of Cao and Li). By leveraging different datasets, we were able to enhance the scope and depth of our analysis, capturing a broader representation of cell populations and developmental stages in the mouse cardiac system. Cao’s whole mouse embryo dataset contains the sequencing results for 2 million cells (GSE119945). Remarkably, Cao proved that these cells have small individual variations. From this dataset, we isolated 2678 cells that had their cardiac muscle lineages labeled by previous research. By employing UMAP visualization, we identified distinct clusters of these cells that reflected their developmental stages (Figure 2A,B). To validate the accuracy of our classification, we compared the assigned labels with the original dataset (Figure 2C) [23]. For the identification of mouse CMs-A and CMs-V, we employed the same marker set as that used in the human dataset. Interestingly, in the mouse cardiomyocytes, these markers exhibited clearer clustering patterns. Specifically, clusters C1 and C3 exhibited characteristics of CMs-A, while C2 and C4 displayed features of CMs-V (Figure 2D). Notably, we also discovered distinct cell populations corresponding to the atrioventricular canal (AVC, C6) and the outflow tract (OFT, C5). The AVC cluster demonstrated higher expression levels of *Rspo3* and *Bmp2*, while the OFT subset exhibited a strong expression of *Rspo3*, which is consistent with previous findings in E10.5 mouse embryonic hearts (Figure 2D,E) [26]. Furthermore, we identified the top 10 DEGs in each cluster, revealing an abundance of *Itga6*, *Nr2f2*, *Tbx5*, and *Tbx18* in CMs-A and *Myh7* and *Myl2* in CMs-V, which aligned with the established markers for these cell types (Figure 2F) [23]. In addition to this, we observed an enrichment of the Prdm16 gene expression in mouse ventricular cardiomyocytes (CMs-V). *Prdm16* is a crucial gene involved in the development of ventricular cardiomyocytes. Remarkably, previous studies identified the deletion of *Prdm16* expression in human embryos with congenital heart disease. Subsequently, they further confirmed this developmental alteration in a mouse model [28,29].

We further examined Cao’s dataset, which comprises mouse endothelial cells from the entire mouse embryo. This subset dataset consists of 25,301 cells that were previously labeled as “endothelial trajectory” [23]. Through UMAP visualization, we assessed the distribution of these cells and their developmental stages (Figure 2G,H). To validate our cell annotations, we referred to the cell annotations from previous studies (Figure 2I) [23]. Notably, since the dataset encompassed endothelial cells from various tissues, we observed a distinct separation of cardiac endothelial cells from cells originating from other tissues. Moreover, we observed that commonly used endothelial cell markers had higher expression levels in non-cardiac ECs compared to cardiac ECs. Specific markers in cardiac ECs (such as *Slc28a2*, *Kcna5*, and *Rftn1*) displayed a more defined expression pattern in mature organisms, rather than during embryonic stages [21] (Figure 2J). By analyzing the top 10 DEGs in each cluster, we identified cluster C5 as cardiac endothelial cells, enriched in the expression of *Gata4*, *Cdh11*, and *Tbx20* (Figure 2K). This cluster consisted of 1925 cells and represented the cardiac endothelial lineage.

Since there is no time-continuous human embryonic single-cell dataset for the heart, we referred here to another mouse embryonic heart dataset as validation material for subsequent experiments. We analyzed Li’s mouse embryonic heart dataset (GSE76118), which covers the developmental stages from E8.5 to E10.5 in mouse embryos. The timing partially overlapped with Cao’s dataset in developmental stages [26]. Through UMAP visualization and unsupervised clustering, we examined the distribution of cells and their corresponding developmental stage (Supplementary Figure S1A,B). Following the same approach as for the human embryonic heart dataset, we filtered out the desired cell types, including CMs-A, CMs-V, and ECs (Supplementary Figure S1C,D). However, it is noteworthy that we observed a high expression level of *FHL2* in the CMs-V of human embryos and the AVC or part of the CMs-A population in mice embryos (Figure 1D and Figure 2D) [14]. *FHL2* is commonly believed to be specifically expressed in adult human CMs-V and plays a significant role in promoting myocardial hypertrophy [30]. When comparing the top 10 DEGs between mouse and human hearts, we found that, in CMs-A, both mouse and human hearts were enriched in *Myh6*/*MYH6* (Figure 1F, Supplementary Figure S1E), while mouse hearts displayed enrichment in *Nr2f2* and human hearts displayed enrichment in *NPPA*, among others (Supplementary Figure S1E). In CMs-V, *Myl2/MYL2* and *Myh7/MYH7* were found to be highly enriched in both mice and humans. Based on these findings, we classified C0 and C5 as CMs-V-like, C1 as CMs-A-like, and C3 as EC-like within the mouse single-cell dataset during heart development.

To gain further insight into cell development, we performed a second-level dimensionality reduction using a 1:1 orthologous gene between humans and mice. By applying this approach, we aimed to identify conserved genes that play significant roles during cell development. The resulting visualization in UMAP allowed us to observe the temporal trends in different cell types during development (Supplementary Figure S2A–F). Interestingly, human embryo cardiomyocytes at 5 weeks showed segregation from other time points; this pattern was not observed in mice (Supplementary Figure S2A–C). But CMs-A and CMs-V from both species exhibited a clear separation and distinct clustering, while cells from different developmental stages did not. This observation suggests a potential difference in the developmental profiles of progenitor cells between the two species.

### 2.3. Global Transcriptome Analysis in Pseudo-Bulk and Identification of Developmentally Dynamic Genes (DDGs)

**Figure 3:**
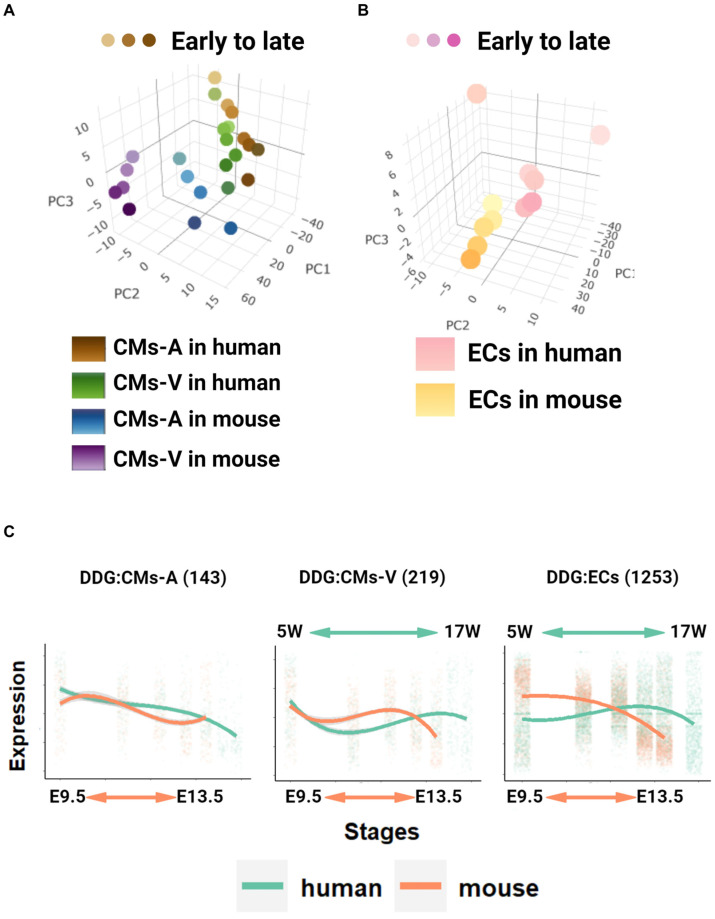
Identification of heart-related developmentally dynamic genes (DDGs). (**A**) A PCA (principal component analysis) conducted based on a 1:1 orthologue across both species delineated CMs-A and CMs-V according to the pseudo-bulk transcriptomes. The shades of color represent the developmental stages of the samples, spanning from 5 to 17 weeks (5W–17W) of gestation in humans and from embryonic day 9.5 to 13.5 in mice. (**B**) As in (**A**), the PCA delineated ECs in both species. (**C**) Expression patterns of DDGs in cardiomyocytes and endothelial cells. From left to right: CMs-A, CMs-V, and ECs. In the graph, each point represents an individual gene, and the lines show cubic spline curves. The different colors denote the species: green for humans and orange for mice. The *x*-axis represents developmental stages on the log2 scale (up: human; down: mouse), while the shaded grey area represents a 95% confidence interval. The *y*-axis represents standardized expression levels of all the genes with a mean of 0 and a standard deviation (SD) of 1. The titles on top of each panel show the gene number in each group. Same as below.

In a pseudo-bulk analysis, the scRNA-seq of cells was combined based on the cell type and the developmental stage, allowing for an exploration of global relationships through a PCA. The pseudo-bulk gene expression densities and hierarchical clustering were used to assess the status of each pseudo-bulk sample. Developmental patterns of cardiomyocytes (CMs) and endothelial cells (ECs) were analyzed separately using a PCA. Variance-stabilizing transformations were applied for visualization. Here, a 3D PCA model revealed distinct global relationships. For CMs, PC1 captured species differences, PC2 reflected ventricle/atrium origin, and PC3 represented developmental stages (Figure 3A). For endothelial cells, PC1 mainly captured species differences, while PC2 and PC3 represented the developmental trajectory (Figure 3B). Incidentally, one outlier sample from the human ECs was removed before PCA visualization.

Then, we investigated DDGs to explore their time-series modifications during developmental stages in gene regulation and organ specificity [31]. We utilized the log-transformed time as the input to identify DDGs in three different cell types (CMs-A, CMs-V, and ECs) between species (human dataset of Cui and mouse dataset of Cao). Our analysis revealed significant cardiac developmental properties in both cell types. We performed an enrichment analysis using the gene ontology (GO) terms for biological processes (BPs) and molecular function (MF). We found that CMs-A exhibited GO enrichment in muscle hypertrophy and development. On the other hand, the involvement of SMAD binding and signaling pathways was associated with *NKX2-5* and TGF-β/BMP in atrial development (Supplementary Figure S3A) [32]. Similarly, CMs-V showed an enrichment of muscle mitotic cells, indicating a focus on cell proliferation in ventricular cardiomyocytes (Supplementary Figure S3B) [33]. Additionally, ECs showed the WNT signaling pathway, highlighting its importance in endothelial cell development, differentiation, and nutrient provision during development (Supplementary Figure S3C) [34].

Previous studies have identified DDGs specific to cardiac development (heart bulk tissues) [4], and we compared this list with the DDGs we identified for the different cell lines of the heart (heart scRNA-seq). Our analysis revealed a statistically significant correlation (hypergeometric test, *p*-value of <0.001) between the DDGs of cardiac cell types and the DDGs identified in the heart bulk tissues of previous studies (Supplementary Figure S3D) [4]. In addition, we investigated the expression patterns of DDGs in different species over time. Notably, we observed distinct trends in humans and mice, with a rising and then falling expression pattern in both datasets (Figure 3C). To validate the reproducibility and continuity of these DDG expressions, we validated them in two mouse development heart datasets (dataset of Cao and Li) with partially overlapping time points, which confirmed similar expression patterns (Supplementary Figure S3E). Our analysis revealed the correlation between DDGs in different cardiac cell types and those identified in bulk tissue samples, highlighting their functional relevance and organ specificity. Furthermore, the distinct expression patterns observed between humans and mice underscored the importance of aligning developmental stages across species.

### 2.4. Stage Correspondence across Species with Dynamic Time Warping (DTW)

**Figure 4:**
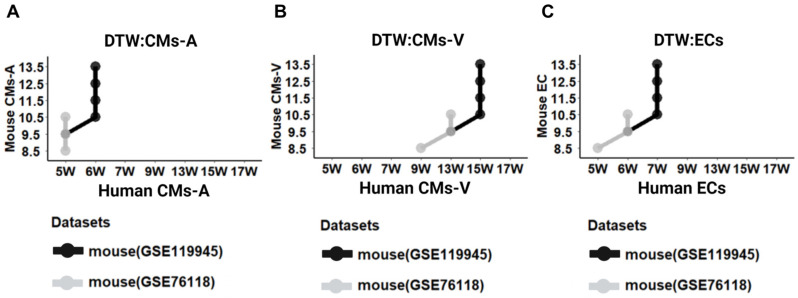
The alignment of dynamic time warping (DTW) between humans and mice to compare developmental stages. (**A**) Comparison of the stage correspondence based on DTW alignment in CMs-A between humans and mice. (**B**) DTW alignment in CMs-V. (**C**) DTW alignment in ECs.

Cross-species cardiac-specific DDGs allowed us to compare and align their expression patterns over time (human: GSE106118; mouse: GSE119945). We aimed to align the developmental stages of CMs-A, CMs-V, and ECs in mice and humans using DTW. Building on previous studies that employed DTW to align heart tissues across species, we utilized single-cell transcriptome-generated pseudo-bulk data to align the developmental stages of different cell types of human and mouse hearts. Our findings, depicted in Figure 4A–C, revealed that the development at 9.5 to 13.5 days in mice corresponded to that at 5 to 6 weeks in humans for CMs-A (Figure 4A, black line), and the development at 9.5 to 13.5 days in mice corresponded to that at 13 to 15 weeks in humans for CMs-V (Figure 4B, black line). Additionally, mouse embryonic cardiac ECs from days 9.5 to 13.5 aligned with 6 to 7 weeks in humans (Figure 4C, black line).

To validate the reliability and robustness of our DTW alignment results, we used another mouse heart dataset to align with the human heart dataset (human: GSE106118; mouse: GSE76118). First, we characterized the second DDGs on heart development in the same way as before (see Section 4). Then, we removed the genes that were duplicated from the previous DDGs. For the GO terms of the cell components (CCs), BPs, and MF in CMs-A, we observed a functional enrichment in muscle myosin (Supplementary Figure S4A), while in CMs-V, we observed enrichment related to the morphogenesis and development of ventricular cardiac muscle (Supplementary Figure S4B). ECs showed enrichment of the GO terms related to aortic formation and the development and differentiation of endothelial cells (Supplementary Figure S4C). Through the use of the second DDGs as input, the DTW results revealed a strong resemblance between the phase alignment of the first and second datasets, with only one phase difference (Figure 4A–C, grey line). It is worth noting that, due to the limited data on human heart embryos, we were unable to evaluate time points before 4 weeks. To ensure accurate comparisons during the CMs-A DTW analysis, DTW was used to align the E8.5 day in mice with the nearest 5 weeks. Remarkably, we observed only one period difference between different batches at the single-cell level, which surpassed the inter-sample variability depicted in the results of the DTW alignment of the bulk heart tissue [4]. These findings indicate that the single-cell results are relatively pure compared to the bulk results derived from whole heart tissue consisting of mixed cell types, supporting the reliability and robustness of the DTW alignment method and reinforcing the validity of the findings.

To further validate our DTW results, we examined the temporal expression patterns of CMs-A, CMs-V, and EC markers in both mouse and human embryos. The expression levels were standardized, and a logarithmic model was applied to represent human developmental time. Previous studies have demonstrated that *KRT8* and *KRT18*, expressed in early cardiomyocytes, decrease over time during human heart development [18]. Consistent with these findings, our pseudo-bulk analysis showed a similar trend, except for mice CMs-A, where Krt8 did not exhibit a decline in expression (Supplementary Figure S4D). Furthermore, we investigated the expression of important genes involved in atrial development, such as *NKX2-5*, *HAND2*, *GATA4*, *NPPA*, *MYL7*, and *NR2F1*. We also analyzed specific markers for CMs-V (*NKX2-5*, *HAND1*, *GATA4*, *NPPB*, *MYL3*, and *LBH*) and ECs (*PECAM1*, *CDH5*, *KDR*, *VWF*, *ELN*, and *EMCN*). The cardiac regulatory genes *GATA4* and *NKX2-5* are recognized as key factors that are activated during both first heart field (FHF) and second heart field (SHF) development, preceding the formation of ventricles and atria. These genes play critical roles in orchestrating the complex processes involved in cardiogenesis. Remarkably, these markers exhibited similar expression patterns across species in a time series under the DTW alignment (Supplementary Figure S4E). Although there were differences in gene expression between humans and mice due to variations in heart development, our alignment of key genes revealed notable similarities. Our findings underscore the feasibility and accuracy of aligning the developmental stages of cardiomyocytes and endothelial cells between mice and humans using DTW, providing insight for the intricate regulatory networks underlying cardiac development.

### 2.5. Differences in Transcription Factors of Different Species of Cardiomyocytes in Atria and Ventricles

**Figure 5:**
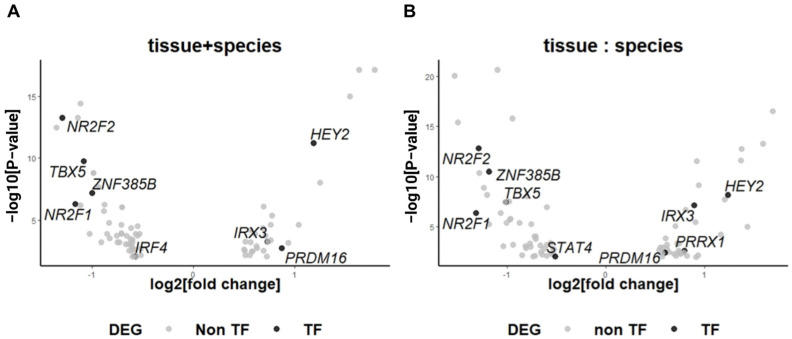
Transcription factors in DEGs and the combined analysis with the *Prdm16^cKO^* cardiomyocytes in mice. The volcano plots present the DEGs in cardiomyocytes. The *x*-axis corresponds to fold changes measured on a log2 scale. The *y*-axis corresponds to the *p*-value measured on a −log10 scale. (**A**) The impacts of the species in cardiomyocyte development. (**B**) The impacts of the species in cardiomyocyte development when controlling for the effect of chambers.

In cardiomyocyte development over time, we hypothesized that some of the transcription factors play a very important role in interspecies variability. In our study, we identified temporally differentially expressed transcription factors in cardiomyocytes from various species and heart chambers. These transcription factors, exhibiting a log2 fold change ≥0.5 and an adj. *p*-value < 0.01, play crucial roles in cardiomyocyte development (Figure 5A,B). Among them, *Prdm16* (PR domain-containing 16) stands out as a gene expressed specifically in the ventricles of embryonic hearts in both humans and mice in a conservative way. Extensive research has linked *Prdm16* to left ventricular non-compaction (LVNC) and dilated cardiomyopathy (DCM) [35,36]. In mouse models, *Prdm16* expression is associated with heart failure, LVNC, morphological defects, and perinatal lethality. Moreover, *Prdm16* directly regulates critical cardiac transcription factors, including *Hey2*, and exhibits an expression correlation with the TGF-β signaling pathway, *Myh7*, and *Nppa*, among others [29].

We observed comparable expression levels of *Prdm16* in CMs-A between humans and mice, while its expression in CMs-V was significantly higher in mice than in human embryos (Supplementary Figure S5A). By using the *Prdm16^cKO^* scRNA-seq dataset (Wu’s, GSE179393; see Table 1) from mouse embryonic hearts and performing a PCA (see Methods), we identified batch effects and species differences (PC1 to PC2) (Supplementary Figure S5B) [29]. By constructing 3D PCA models based on PC3 to PC5, we visualized the developmental trajectories of cardiomyocytes (Supplementary Figure S5C). The global PCA indicated that the trajectory of human cardiomyocytes was central, while the mouse trajectory occupied the sides. Notably, the trajectory exhibited by the CMS-V in *Prdm16^cKO^* mice was closer to that of human cardiomyocytes (Supplementary Figure S5C). The t-distributed stochastic neighbor embedding (t-SNE) highlighted the differences across the species, and in the *Prdm16^cKO^* mice, CMs-V resembled those of humans more closely (Supplementary Figure S5D). These findings suggest that differential *Prdm16* expression levels in CMs-V may contribute to species-specific differences between humans and mice.

Given the differential expression of *Prdm16* between species and its role as a critical transcription factor in cardiomyocyte development, we investigated its downstream transcriptional binding sites. A previous *Prdm16^cKO^* study revealed that a knockout resulted in DEGs in CMs-V, and ChIP-seq experiments validated the direct regulatory genes of *Prdm16* [29]. Remarkably, we observed a significant correlation between the DEGs detected in various species and heart chambers with the DEGs identified in the CMs-V of *Prdm16^cKO^* mice, as well as the genes directly targeted by *Prdm16*. These findings confirm our initial hypothesis and are supported by the results of hypergeometric tests (*p*-value < 0.0001) (Supplementary Figure S5E).

### 2.6. Joint Analysis with the Chamber-like Cardiomyocytes Derived from Human Pluripotent Stem Cells (hPSCs)

The investigation of in vitro differentiated cardiomyocytes derived from hPSCs and their similarities to naturally grown cardiomyocytes in vivo is an important field of cardiovascular research, with implications for heart disease therapy. Single-cell transcriptomes provide us with great help in comparing similarities between in vivo and in vitro cardiomyocytes. Previous studies have shown promise in creating chamber-specific cardiomyocytes from hPSCs that closely resemble in vivo characteristics [37].

**Figure 6:**
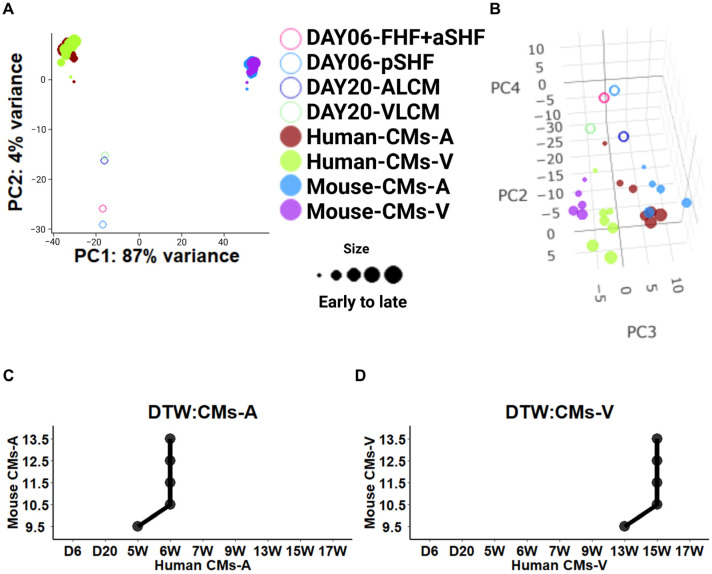
The combined analysis with cardiomyocytes from different hPSC-derived chambers and dynamic time warping (DTW). (**A**) A PCA based on a 1:1 orthologue within the datasets of embryonic heart cardiomyocytes and hPSC-derived cardiomyocytes. The origin of cardiomyocytes is represented by different shapes, and the developmental stages are indicated by the sizes of the dots. (**B**) The 3D PCA, showing PCs 3, 4, and 5, with the same characteristics as in (**A**). (**C**) The stage correspondence was compared based on the DTW alignment in atrial cardiomyocytes (CMs-A) between the two species. hPSC-derived cardiomyocytes were added preceding the embryonic stage. The same was performed in (**D**). (**D**) DTW alignment in CMs-V.

In our study, we included a subset of in vitro cultured chamber-specific cardiomyocytes in our pseudo-bulk developmental dataset (Yang’s dataset, GSE173486). By selecting chamber-like cardiomyocytes from the single-cell RNA dataset, we identified that the day 6 and day 20 developmental time points of Yang’s dataset exhibited chamber-specific cardiomyocytes (see Methods, Section 4). We performed a PCA to differentiate samples by species (PC1) and developmental stages (PC2) (Figure 6A). A further analysis combining PC2, PC3, and PC4 allowed us to identify samples based on the chambers and developmental stages, providing insight into the distinct characteristics of cardiomyocytes derived from different chambers by global PCA (Figure 6B). PC2 explained the difference in the period of cardiomyocyte development. Interestingly, we observed that the chamber-specific cardiomyocytes derived from hPSCs were at an earlier developmental stage, close to the earliest samples from the in vivo chambers [24,38,39]. PC3 explained the difference between the atrial and ventricular cardiomyocytes in vivo and in vitro. This suggests that they express a strong commonality, and we speculate that their developmental programs are still largely shared across the lineage.

We aimed to investigate whether there is a correlation between the developmental stage of mouse embryonic cardiomyocytes and hPSC-derived cardiomyocytes. We positioned the time points of hPSC-derived cardiomyocytes before the 5-week time point in human embryos based on the developmental pattern derived from the PCA. We assumed here that the in vitro model of differentiated cardiomyocytes was in a more immature state prior to the 5-week-old cardiomyocytes of a human embryo. We then identified cross-species DDGs related to heart development and the GO analysis. The analysis revealed the enrichment of GO terms related to the cardiac muscle, cardiac process, and muscle development, indicating the similarity of gene expression patterns between mouse and human cardiomyocytes during development (Supplementary Figure S6A,B). The fitted curve of the cubic model shows the expression profile of these DDGs (Supplementary Figure S6C). We found that, in human embryonic cardiomyocytes, their expression presumably undergoes a decreasing trend (CMs-A, S6C top, green curve), or decreases and then increases (CMs-V S6C, bottom, green curve). Interestingly, we found that the expression levels of these DDGs were largely decreased from day 6 to day 20 in hPSC-derived cardiomyocytes. In addition to this, we observed that the expression levels of these DDGs were higher in the in vitro cardiomyocytes than in the embryonic cardiomyocytes. This is consistent with our hypothesis about cardiomyocyte maturation and gives us an approximate transcriptomic idea of the disparity in the extent of cardiomyocyte development in vivo and in vitro (Supplementary Figure S6C).

To identify whether mouse embryonic cardiomyocytes are matchable to hPSC-derived cardiomyocytes and to further validate the reliability of our previous DTW results, we proceeded to align cardiomyocytes from both species by DTW. The DTW results showed no difference from the previous cross-species alignment results, in which the development at 9.5 to 13.5 days in mice corresponded to that at 5 to 6 weeks in humans for CMs-A and the development at 9.5 to 13.5 days in mice corresponded to that at 13 to 15 weeks in humans for CMs-V (Figure 6C,D). This demonstrates that the similarity between mouse cardiomyocytes and hPSC-derived cardiomyocytes is weaker than the similarity between mouse and human embryonic cardiomyocytes. We speculate that this is due to the substantial gap between day 20 cardiomyocytes cultured in vitro and human cardiomyocytes at 5 weeks (Figure 6C,D).

We elaborated on the results of previous research by investigating the relationship among pSHF progenitors that give rise to atrial-like CMs (ALCMs)^(*NR2F2*+,*CAV1*+,*NKX2−5*+)^ and the embryonic CMs-A in their expression [37]. In hPSC-derived ALCMs, the markers that are specifically expressed are *NR2F2*, *CAV1*, and *NKX2-5*. We showed the expression pattern of human and mouse embryonic CMs-A based on DTW alignment correspondence (Supplementary Figure S6D). During the maturation of ALCMs, the average expression levels of *NKX2-5* and *CAV1* increased, whereas we found that the expression levels in the CMs-A of human embryos were at a higher level here. Similarly, NR2F2 showed a decreasing trend in expression with maturation, whereas the expression in CMs-A was lower in human embryos.

A previous study identified 142 expressed genes that were conserved with hPSC-derived atrial cardiomyocyte expression in a mouse embryo at E9.25 [37]. On this basis, we imported CMs-A from our human and mouse embryos and observed their expression changes over time (Supplementary Figure S6E). Interestingly, we observed that this group of genes showed a trend of increasing and then decreasing in human CMs-A, whereas in mice, the trend showed a general decrease. In comparison, these 142 genes with a conserved expression were shown to rise in hPSC-derived atrial cardiomyocytes with developmental expression over time. We hypothesize that this flanks the consistency of the human in vivo and in vitro myocardial lineage and the differential expression of this fraction of genes across species.

## 3. Discussion

Differences in organ development among species are usually associated with changes in gene expression. Based on previous studies, at the organ level, the brain showed the least variation in development, while the liver and testes showed the highest level of variation, and the variation in heart development was in between [3,4]. DTW was applied to align the cardiac organ developmental stages across species [4,17]. In a single-cell analysis, DTW was applied to cross-species comparisons to align the developmental stages of neuronal cells from different species [22]. In bulk RNA-seq studies of the heart, an approximate understanding of the stages of cardiac development among different species has been obtained [4]. In heart single-cell analyses, previous studies have compared part of the cell expression profiles across species [7,9,18]. However, the expression level of the cross-species transcriptome at the single-cell level in the time series of heart development is unclear. In this exploratory, cross-species study, we examined cardiomyocytes from different subpopulations and endothelial cells at the single-cell level. The dynamic time-warping (DTW) analysis revealed distinct results when considering varying degrees of transcriptome sequencing. This analysis allowed for the identification of potential cross-species cardiac-development-related genes expressed in different cell populations. We found in this study that humans and mice are likely to share programs for myocardial development in factors such as *NKX2-5* and *GATA4*. Possible cross-species developmental genes in different cell types were also labeled (Supplementary Figure S6F). By examining the transcriptomic profiles across species, we gained insight into the shared genetic regulatory networks involved in cardiac development, elucidating the conserved mechanisms underlying this complex process.

We found different results for the different chambers of developmental stages between species, even in the same cell type (cardiomyocytes). Overall, in CMs-A and ECs, these results are similar to the previous DTW results of bulk-seq, which showed that cardiac-specific correspondences were consistent with global correspondences [4]. On the other hand, the inconsistency in the correspondence of developmental stages between the different lineages was also confirmed. We found different results for CMs-V versus CMs-A in the single-cell sequencing results of cardiomyocyte development based on chamber division. From an organizational perspective, the atria of humans are prominent structures, while in the mouse heart, the atrial chambers are relatively small. Ventricular trabeculation provides 80% of the myocardial mass before ventricular separation during realistic human and mouse heart development [40]. After the formation of the ventricular septum, trabeculae begin to compact. Previous studies have shown this process to be rapid, with day E13 in mice being equivalent to 12 to 14 weeks in humans [6,41]. This suggests that, perhaps during the development of ventricular cardiomyocytes, additional molecular regulatory mechanisms may exist in humans and mice for the development of cardiomyocytes. This hypothesis was verified in later explorations of *Prdm16*. Further, the discrepancies observed between different datasets in our study were significantly smaller than those encountered in bulk RNA-seq approaches, highlighting the advantageous nature of a single-cell analysis [4]. Based on the findings of this study, we significantly enhanced our comprehension of the role of mouse animal experiments in heart-related drug trials. Furthermore, we gained a better understanding of the distinctions between mouse and human embryos in models of congenital heart disease. By bridging the knowledge gap, we can optimize the design and interpretation of studies utilizing mouse models, thereby advancing our understanding of cardiac pathologies and improving the translational potential of clinical research, which will lead to the better clinical diagnosis and treatment of heart diseases [5,6].

We applied a pseudo-bulk paradigm to allow our single-cell transcriptome datasets to ignore cell-to-cell variation and focus more on the variability across species, heart chambers, in vivo studies, and in vitro studies. On this basis, we obtained chamber-specific cardiomyocytes from previous studies. We determined the gap between them and embryonic cardiomyocytes in part using a joint analysis. In Cao’s dataset, the samples were collected from whole mouse embryos, and we made every effort to segregate cardiomyocytes and endothelial cells from heart tissue. However, due to challenges in discerning their tissue origin, we encountered difficulties in extracting cell types such as fibroblasts and immune cells in heart tissue. Consequently, we do not currently have alignment results for these specific cell types. Furthermore, for various reasons, the scarcity of temporally continuous embryonic samples in the available datasets has precluded our discovery of a single-cell dataset that encompasses spatial transcriptomic features.

Moving forward, our research aims to broaden the scope by encompassing additional cell types, expanding the coverage across various developmental stages, and enhancing the spatial anatomical resolution. Because non-coding RNAs (ncRNAs) are highly involved in genomic regulatory functions and exhibit a strong cellular specificity, in the future, we will also explore their role in organ development and evolution.

## 4. Materials and Methods

### 4.1. Identification of Cell Types

In single-cell datasets (human heart: GSE106118, mouse embryo: GSE119945, mouse heart: GSE76118, see Table 1), the gene expression levels were quantified as transcripts per million (TPM). The TPM values were converted to log2 values (TPM + 1). The processed expression data were used to identify the cell types. In quality control, we established thresholds for the number of features. For human samples, we set a limit of nFeature > 1000, while for mouse samples, the limit was nFeature > 600. These thresholds helped to ensure the accuracy and reliability of our findings. The Seurat R package was used to select 18 PCs for the UMAP analysis [42]. Cell clusters were then made by setting the clustering parameter resolution of the FindClusters function in Seurat from 0.3 to 0.8. Dotplots with cell-type-specific marker expression patterns were visualized using jjDotPlot commands in scRNAtoolVis package [43]. The rest of the visualizations (heatmap, featureplot) were performed in this package.

### 4.2. Construction of Pseudo-Bulk Matrix and Execution of PCA

We used biomaRt to identify 1:1 orthologs between the species [44]. AverageExpression in Seurat was used to construct an expression matrix of several stages in different species by time and cell type. The resulting value was added to 1, multiplied by 10,000, and rounded to become an integer. The sample expression distribution curves were evaluated with the help of the plotDensities function [45]. The global and organ-specific PCA read counts were used as the input after applying the variance-stabilizing transformation (VST) implemented in the DESeq2 package [46]. In the PCA, all pseudo-bulk samples underwent unsupervised hierarchical clustering (hclust) to evaluate the quality confirmation outlier status. The coordinates in PCs were obtained using a modified PCA plot, and a 3D image was constructed in the plotly package [47].

### 4.3. Developmentally Dynamic Genes (DDGs)

The MaSigPro package was used to identify developmentally dynamic genes (DDGs) in atrial cardiomyocytes (CMs-A), ventricular cardiomyocytes (CMs-V), and endothelial cells (ECs) for both species [48]. We ran maSigPro on log-transformed times (measured in days of gestation) in degrees = 3 (polynomial) and considered genes as DDGs in organs when the goodness of fit (R2) was at least 0.3. For each cell type, we identified clusters of these genes by hierarchical clustering (hclust). Clusters containing terms related to cardiac development were selected with the help of the enrichGO command of the clusterProfiler package [49]. Finally, we used the hypergeometric test in the GeneOverlap package to display the correlation between the DDGs identified in this study and the DDGs in the whole heart tissue of previous research [50]. The second batch of DDGs in the three cell types (CMs-A, CMs-V, and ECs) was identified in the human embryonic heart (Cui’s dataset) and another mouse embryonic heart dataset (Li’s dataset), and then the genes that overlapped with the first batch were removed.

### 4.4. Stage Correspondence across Species in Dynamic Time Warping (DTW)

DTW was run using 1:1 DDGs corresponding to cell types to identify the stage correspondence across species. We aligned the mouse developmental stages to those in humans (after log-transforming the days) using the “align_pt_traj” function to a degree equal to 3 and a partial mode among CMs-A, CMs-V, and ECs [22]. The human time points were expressed as a reference, and the points in one time series were matched with the points in another time series. The age order of the species was maintained and the distance between the resulting curves was calculated. The system automatically selected the alignment that produced the smallest distance between the curves. Curve-fitting graphics were obtained in ggplot2, with the time axis as the log time of human embryos by fitting a cubic polynomial formula [51].

### 4.5. The Transcription Factor (TF) in Differentially Expressed Genes (DEGs)

DESeq2 was used to identify DEGs in cardiomyocytes that were differentially expressed between species and chambers [46]. The adjusted *p*-value was required to be ≤0.01, and the log2 fold change was required to be ≥0.5. A list of transcription factors was obtained from the animalTFDB database [52]. The t-SNE was mapped in the Rtsne package on a VST-transformed matrix with a perplexity of 8 [53].

### 4.6. Combining Analysis between Prdm16^cKO^ Mouse Heart and hPSC-Derived Cardiomyocytes

In single-cell datasets (Wu’s *Prdm16^cKO^*: GSE179393, Yang’s hPSC: GSE173486, see Table 1), we continuously used log2 (TPM + 1) to transform the raw expression matrix before the quality control and the dimensional reduction. In the *Prdm16^cKO^* dataset, we selected the CMs-V (MYH7+, MYL2+) of E13.5-day WT and *Prdm16^cKO^* mouse embryos with reference to the original study [29]. These cells were then combined to generate pseudo-bulk samples. In the hPSC dataset, we selected their atrial-like myocardium (ALCM) and ventricular-like myocardium (VLCM) separately. To be more specific, we referred to the original study to question the cell labels and expression of specific markers. In the day 6 samples, we isolated the progenitor cell populations of the first heart field (FHF), the anterior second heart field (aSHF), and the posterior second heart field (pSHF). Moreover, the pSHF was classified as ALCM, while the FHF and aSHF were classified as VLCM as described by YANG [37]. On day 20, we took into account the differentiation efficiency as well as the cell purity. After clustering, we first removed the sinus venosus and OFT CMs based on the special markers mentioned in their own study. The remaining cells were merged into pseudo-bulk patterns in the ALCM and VLCM models. The PCA inputs were shown in a VST-transformed matrix. MaSigPro was run on the log-transformed days extracted from the hPSC culture merged with heart development in the human embryonic heart to extract the DDGs. DTW was then applied to these DDGs. The list of 142 conserved atrial cardiomyocyte (ACM) genes from previous research was used [37].

## 5. Conclusions

Through our investigation into the disparities between humans and mice in cardiac development at the single-cell level, we obtained a deeper understanding of the cross-species heterogeneity within different cell populations of the heart. Notably, our findings revealed distinct developmental outcomes in cardiomyocytes between the atria and ventricles, indicating the presence of diverse regulatory mechanisms governing chamber development across species. *Prdm16* has been shown to play an important role in ventricular development in previous studies. In our results, we found different levels of its expression across species, further illustrating the heterogeneity of cardiomyocyte development between humans and mice. Furthermore, our study encompasses a comparative analysis between in vitro induced differentiated cardiomyocytes and embryonic cardiomyocytes. While acknowledging the existence of certain disparities between these two cell populations, it is undeniable that they exhibit a general chamber specificity. This intriguing finding provides valuable insight into the potential applications of in vitro cultured cardiomyocytes, offering an opportunity to deepen our understanding of cardiac development and advance therapeutic strategies for cardiac ailments. Moreover, these discoveries establish a robust foundation for investigating the applicability of mouse models in heart disease research, enabling comprehensive investigations into disease mechanisms and interventions at a more detailed level.

## Figures and Tables

**Table 1:** Datasets used in this study.

GEO Accessibility	First Author Name	Heart Region/Cell Type	Time Points
GSE106118 (human)	Yueli Cui	Whole heart	5 weeks (5 W) to 25 W
GSE119945 (mouse)	Junyue Cao	Whole heart and other organs	E9.5 to E13.5 days
GSE76118 (mouse)	Guang Li	Whole heart	E8.5 to E10.5 days
GSE179393 (mouse)	Tongbin Wu	Whole heart (*Prdm16^cKO^*)	E13.5 days
GSE173486 (hPSCs)	Donghe Yang	hPSC-induced cardiomyocytes	Day 6, day 20

## Data Availability

All the datasets in this study were obtained from public databases. [dataset] Yuxuan Zheng. 2019. Single-cell transcriptome analysis maps the developmental track of the human heart; GEO; GSE106118. [dataset] Junyue Cao. 2019. The dynamic transcriptional landscape of mammalian organogenesis at single-cell resolution; GEO; GSE119945. [dataset] Guang Li. 2016. Transcriptomic profiling maps anatomically patterned subpopulations among single embryonic cardiac cells [RNA-seq]; GEO; GSE76118. [dataset] Ju Chen. 2021 Prdm16 is a compact myocardium-enriched transcription factor required to maintain compact myocardial cardiomyocyte identity in left ventricle; GEO; GSE179393. [dataset] Gordon Keller. 2022 Single-cell transcriptomic profiles of hPSC-derived cardiac cells of various lineages and stages; GEO; GSE173486.

## Supplementary Materials

The relevant operating code as well as the configuration files can be found at https://github.com/MedCYLIU/Heart_development_DTW.git (accessed on 20 September 2023). The following supporting information can be downloaded at: https://www.mdpi.com/article/10.3390/ijms25063240/s1.

## References

[1] Savoji H., Mohammadi M.H., Rafatian N., Toroghi M.K., Wang E.Y., Zhao Y., Korolj A., Ahadian S., Radisic M. (2019). Cardiovascular Disease Models: A Game Changing Paradigm in Drug Discovery and Screening. Biomaterials.

[2] Dickinson M.E., Flenniken A.M., Ji X., Teboul L., Wong M.D., White J.K., Meehan T.F., Weninger W.J., Westerberg H., Adissu H. (2016). High-Throughput Discovery of Novel Developmental Phenotypes. Nature.

[3] Brawand D., Soumillon M., Necsulea A., Julien P., Csárdi G., Harrigan P., Weier M., Liechti A., Aximu-Petri A., Kircher M. (2011). The Evolution of Gene Expression Levels in Mammalian Organs. Nature.

[4] Cardoso-Moreira M., Halbert J., Valloton D., Velten B., Chen C., Shao Y., Liechti A., Ascenção K., Rummel C., Ovchinnikova S. (2019). Gene Expression across Mammalian Organ Development. Nature.

[5] Krishnan A., Samtani R., Dhanantwari P., Lee E., Yamada S., Shiota K., Donofrio M.T., Leatherbury L., Lo C.W. (2014). A Detailed Comparison of Mouse and Human Cardiac Development. Pediatr. Res..

[6] Wessels A., Sedmera D. (2003). Developmental Anatomy of the Heart: A Tale of Mice and Man. Physiol. Genom..

[7] Anzai T., Yamagata T., Uosaki H. (2020). Comparative Transcriptome Landscape of Mouse and Human Hearts. Front. Cell Dev. Biol..

[8] Gao J., Zheng Y., Li L., Lu M., Chen X., Wang Y., Li Y., Liu X., Gao Y., Mao Y. (2021). Integrated transcriptomics and epigenomics reveal chamber-specific and species-specific characteristics of human and mouse hearts. PLoS Biol..

[9] Shang M., Hu Y., Cao H., Lin Q., Yi N., Zhang J., Gu Y., Yang Y., He S., Lu M. (2022). Concordant and Heterogeneity of Single-Cell Transcriptome in Cardiac Development of Human and Mouse. Front. Genet..

[10] Basu M., Garg V. (2018). Maternal Hyperglycemia and Fetal Cardiac Development: Clinical Impact and Underlying Mechanisms. Birth Defects Res..

[11] Brade T., Pane L.S., Moretti A., Chien K.R., Laugwitz K.-L. (2013). Embryonic Heart Progenitors and Cardiogenesis. Cold Spring Harb. Perspect. Med..

[12] de Boer B.A., van den Berg G., de Boer P.A.J., Moorman A.F.M., Ruijter J.M. (2012). Growth of the Developing Mouse Heart: An Interactive Qualitative and Quantitative 3D Atlas. Dev. Biol..

[13] Savolainen S.M., Foley J.F., Elmore S.A. (2009). Histology Atlas of the Developing Mouse Heart with Emphasis on E11.5 to E18.5. Toxicol. Pathol..

[14] Litviňuková M., Talavera-López C., Maatz H., Reichart D., Worth C.L., Lindberg E.L., Kanda M., Polanski K., Heinig M., Lee M. (2020). Cells of the Adult Human Heart. Nature.

[15] Zhou P., Pu W.T. (2016). Recounting Cardiac Cellular Composition. Circ. Res..

[16] Pinto A.R., Ilinykh A., Ivey M.J., Kuwabara J.T., D’Antoni M.L., Debuque R., Chandran A., Wang L., Arora K., Rosenthal N.A. (2016). Revisiting Cardiac Cellular Composition. Circ. Res..

[17] Liu X., Somel M., Tang L., Yan Z., Jiang X., Guo S., Yuan Y., He L., Oleksiak A., Zhang Y. (2012). Extension of Cortical Synaptic Development Distinguishes Humans from Chimpanzees and Macaques. Genome Res..

[18] Cui Y., Zheng Y., Liu X., Yan L., Fan X., Yong J., Hu Y., Dong J., Li Q., Wu X. (2019). Single-Cell Transcriptome Analysis Maps the Developmental Track of the Human Heart. Cell Rep..

[19] Wang Y., Yao F., Wang L., Li Z., Ren Z., Li D., Zhang M., Han L., Wang S., Zhou B. (2020). Single-Cell Analysis of Murine Fibroblasts Identifies Neonatal to Adult Switching That Regulates Cardiomyocyte Maturation. Nat. Commun..

[20] Gawronski K.A.B., Kim J. (2017). Single Cell Transcriptomics of Noncoding RNAs and Their Cell-Specificity. Wiley Interdiscip. Rev. RNA.

[21] Paik D.T., Tian L., Williams I.M., Rhee S., Zhang H., Liu C., Mishra R., Wu S.M., Red-Horse K., Wu J.C. (2020). Single-Cell RNA Sequencing Unveils Unique Transcriptomic Signatures of Organ-Specific Endothelial Cells. Circulation.

[22] Kanton S., Boyle M.J., He Z., Santel M., Weigert A., Sanchís-Calleja F., Guijarro P., Sidow L., Fleck J.S., Han D. (2019). Organoid Single-Cell Genomic Atlas Uncovers Human-Specific Features of Brain Development. Nature.

[23] Cao J., Spielmann M., Qiu X., Huang X., Ibrahim D.M., Hill A.J., Zhang F., Mundlos S., Christiansen L., Steemers F.J. (2019). The Single-Cell Transcriptional Landscape of Mammalian Organogenesis. Nature.

[24] Guo Y., Pu W.T. (2020). Cardiomyocyte Maturation. Circ. Res..

[25] Padula S.L., Velayutham N., Yutzey K.E. (2021). Transcriptional Regulation of Postnatal Cardiomyocyte Maturation and Regeneration. Int. J. Mol. Sci..

[26] Li G., Xu A., Sim S., Priest J.R., Tian X., Khan T., Quertermous T., Zhou B., Tsao P.S., Quake S.R. (2016). Transcriptomic Profiling Maps Anatomically Patterned Subpopulations among Single Embryonic Cardiac Cells. Dev. Cell.

[27] Wilson K.D., Ameen M., Guo H., Abilez O.J., Tian L., Mumbach M.R., Diecke S., Qin X., Liu Y., Yang H. (2020). Endogenous Retrovirus-Derived LncRNA BANCR Promotes Cardiomyocyte Migration in Humans and Non-Human Primates. Dev. Cell.

[28] Cibi D.M., Bi-Lin K.W., Shekeran S.G., Sandireddy R., Tee N., Singh A., Wu Y., Srinivasan D.K., Kovalik J.-P., Ghosh S. (2020). Prdm16 Deficiency Leads to Age-Dependent Cardiac Hypertrophy, Adverse Remodeling, Mitochondrial Dysfunction, and Heart Failure. Cell Rep..

[29] Wu T., Liang Z., Zhang Z., Liu C., Zhang L., Gu Y., Peterson K.L., Evans S.M., Fu X.-D., Chen J. (2022). PRDM16 Is a Compact Myocardium-Enriched Transcription Factor Required to Maintain Compact Myocardial Cardiomyocyte Identity in Left Ventricle. Circulation.

[30] Kong Y., Shelton J.M., Rothermel B., Li X., Richardson J.A., Bassel-Duby R., Williams R.S. (2001). Cardiac-Specific LIM Protein FHL2 Modifies the Hypertrophic Response to β-Adrenergic Stimulation. Circulation.

[31] Pantalacci S., Sémon M. (2015). Transcriptomics of Developing Embryos and Organs: A Raising Tool for Evo–Devo. J. Exp. Zool. Part B Mol. Dev. Evol..

[32] Yuan S.-M., Jing H. (2010). Cardiac Pathologies in Relation to Smad-Dependent Pathways. Interact. CardioVascular Thorac. Surg..

[33] Barak Y., Hemberger M., Sucov H.M. (2019). Phases and Mechanisms of Embryonic Cardiomyocyte Proliferation and Ventricular Wall Morphogenesis. Pediatr. Cardiol..

[34] Manukjan N., Ahmed Z., Fulton D., Blankesteijn W.M., Foulquier S. (2020). A Systematic Review of WNT Signaling in Endothelial Cell Oligodendrocyte Interactions: Potential Relevance to Cerebral Small Vessel Disease. Cells.

[35] Arndt A.-K., Schafer S., Drenckhahn J.-D., Sabeh M.K., Plovie E.R., Caliebe A., Klopocki E., Musso G., Werdich A.A., Kalwa H. (2013). Fine Mapping of the 1p36 Deletion Syndrome Identifies Mutation of PRDM16 as a Cause of Cardiomyopathy. Am. J. Hum. Genet..

[36] Myasnikov R.P., Bukaeva A.A., Kulikova O.V., Ershova A.I., Petukhova A.V., Zotova E.D., Meshkov A.N., Mershina E.A., Kiseleva A.V., Divashuk M.G. (2021). New variant of PRDM16 gene nucleotide sequence in a family with various phenotypic manifestations of the non-compacted myocardium. Russ. J. Cardiol..

[37] Yang D., Gomez-Garcia J., Funakoshi S., Tran T., Fernandes I., Bader G.D., Laflamme M.A., Keller G.M. (2022). Modeling Human Multi-Lineage Heart Field Development with Pluripotent Stem Cells. Cell Stem Cell.

[38] de Soysa T.Y., Ranade S.S., Okawa S., Ravichandran S., Huang Y., Salunga H.T., Schricker A., del Sol A., Gifford C.A., Srivastava D. (2019). Single-Cell Analysis of Cardiogenesis Reveals Basis for Organ-Level Developmental Defects. Nature.

[39] Xu X.Q., Soo S.Y., Sun W., Zweigerdt R. (2009). Global Expression Profile of Highly Enriched Cardiomyocytes Derived from Human Embryonic Stem Cells. Stem Cells.

[40] Jensen B., Agger P., de Boer B.A., Oostra R.-J., Pedersen M., van der Wal A.C., Nils Planken R., Moorman A.F.M. (2016). The Hypertrabeculated (Noncompacted) Left Ventricle Is Different from the Ventricle of Embryos and Ectothermic Vertebrates. Biochim. Et Biophys. Acta (BBA) Mol. Cell Res..

[41] Samsa L.A., Yang B., Liu J. (2013). Embryonic Cardiac Chamber Maturation: Trabeculation, Conduction, and Cardiomyocyte Proliferation. Am. J. Med. Genet. Part C Semin. Med. Genet..

[42] Hao Y., Hao S., Andersen-Nissen E., Mauck W.M., Zheng S., Butler A., Lee M.J., Wilk A.J., Darby C., Zager M. (2021). Integrated Analysis of Multimodal Single-Cell Data. Cell.

[43] Zhang J. (2022). scRNAtoolVis: Useful Functions to Make Your scRNA-seq Plot More Cool!. https://github.com/junjunlab/scRNAtoolVis.

[44] Durinck S., Spellman P.T., Birney E., Huber W. (2009). Mapping Identifiers for the Integration of Genomic Datasets with the R/Bioconductor Package BiomaRt. Nat. Protoc..

[45] Ritchie M.E., Phipson B., Wu D., Hu Y., Law C.W., Shi W., Smyth G.K. (2015). Limma Powers Differential Expression Analyses for RNA-Sequencing and Microarray Studies. Nucleic Acids Res..

[46] Love M.I., Huber W., Anders S. (2014). Moderated Estimation of Fold Change and Dispersion for RNA-Seq Data with DESeq2. Genome Biol..

[47] Sievert C. (2020). Interactive Web-Based Data Visualization with R, Plotly, and Shiny.

[48] Nueda M.J., Tarazona S., Conesa A. (2014). Next MaSigPro: Updating MaSigPro Bioconductor Package for RNA-Seq Time Series. Bioinformatics.

[49] Wu T., Hu E., Xu S., Chen M., Guo P., Dai Z., Feng T., Zhou L., Tang W., Zhan L. (2021). ClusterProfiler 4.0: A Universal Enrichment Tool for Interpreting Omics Data. Innovation.

[50] Shen L., Icahn School of Medicine at Mount Sinai (2021). GeneOverlap: Test and Visualize Gene Overlaps. R Package Version 1.30.0. http://shenlab-sinai.github.io/shenlab-sinai/.

[51] Wickham H. (2016). Ggplot2: Elegant Graphics for Data Analysis.

[52] Zhang H.-M., Liu T., Liu C.-J., Song S., Zhang X., Liu W., Jia H., Xue Y., Guo A.-Y. (2015). AnimalTFDB 2.0: A Resource for Expression, Prediction and Functional Study of Animal Transcription Factors. Nucleic Acids Res..

[53] Jesse H. (2015). Krijthe Rtsne: T-Distributed Stochastic Neighbor Embedding using a Barnes-Hut Implementation. https://github.com/jkrijthe/Rtsne.

