# Supplementary figures and images for: The Differences in the Developmental Stages of the Cardiomyocytes and Endothelial Cells in Human and Mouse Embryos at the Single-Cell Level
Source: Int J Mol Sci. 2024 Mar 13;25(6):3240. doi: 10.3390/ijms25063240 (PMC10970218; doi:10.3390/ijms25063240)

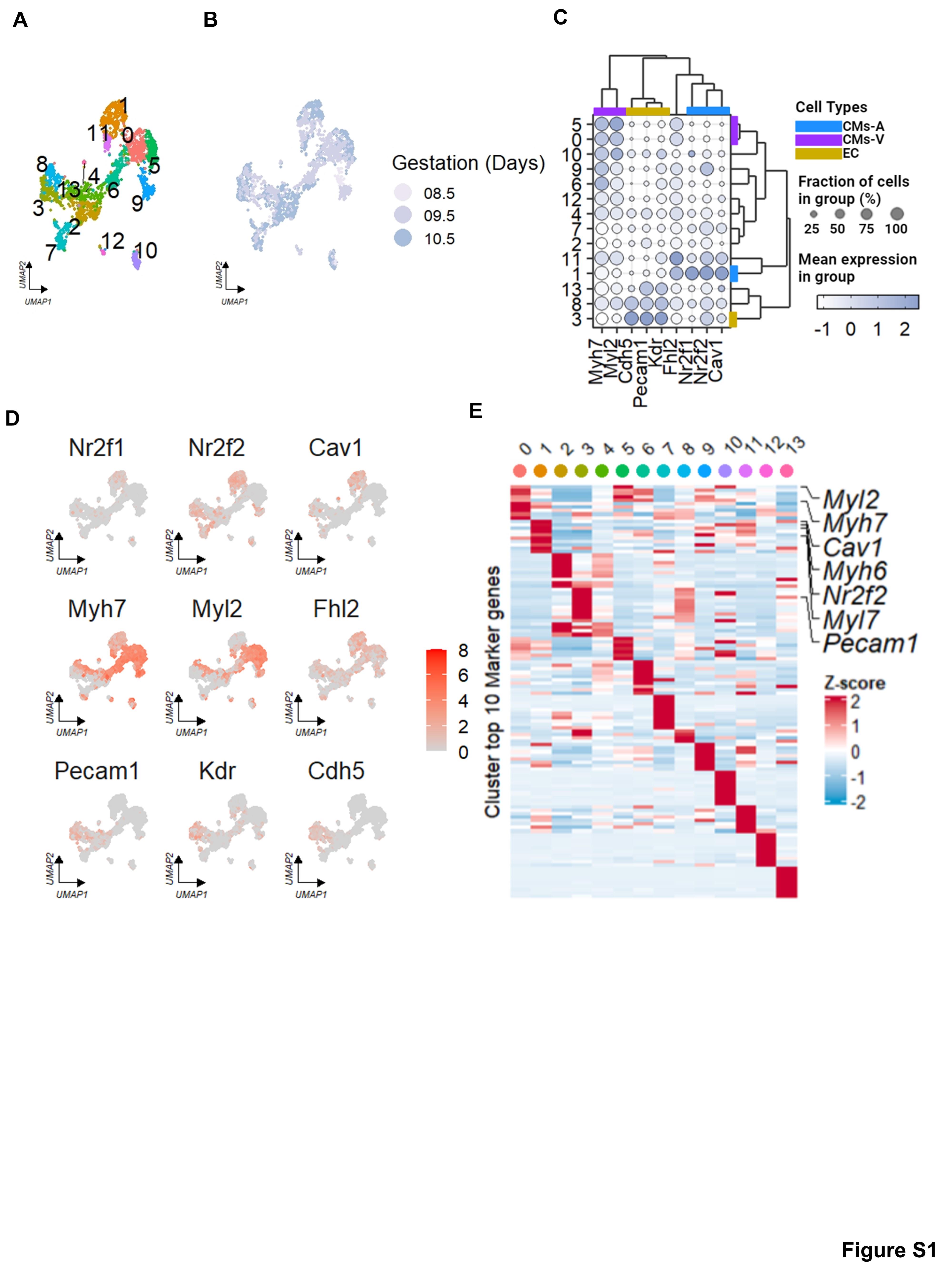

Supplement: Supplementary file 1 [file ijms-25-03240-s001.zip › Supplementary FIG 1.jpg]

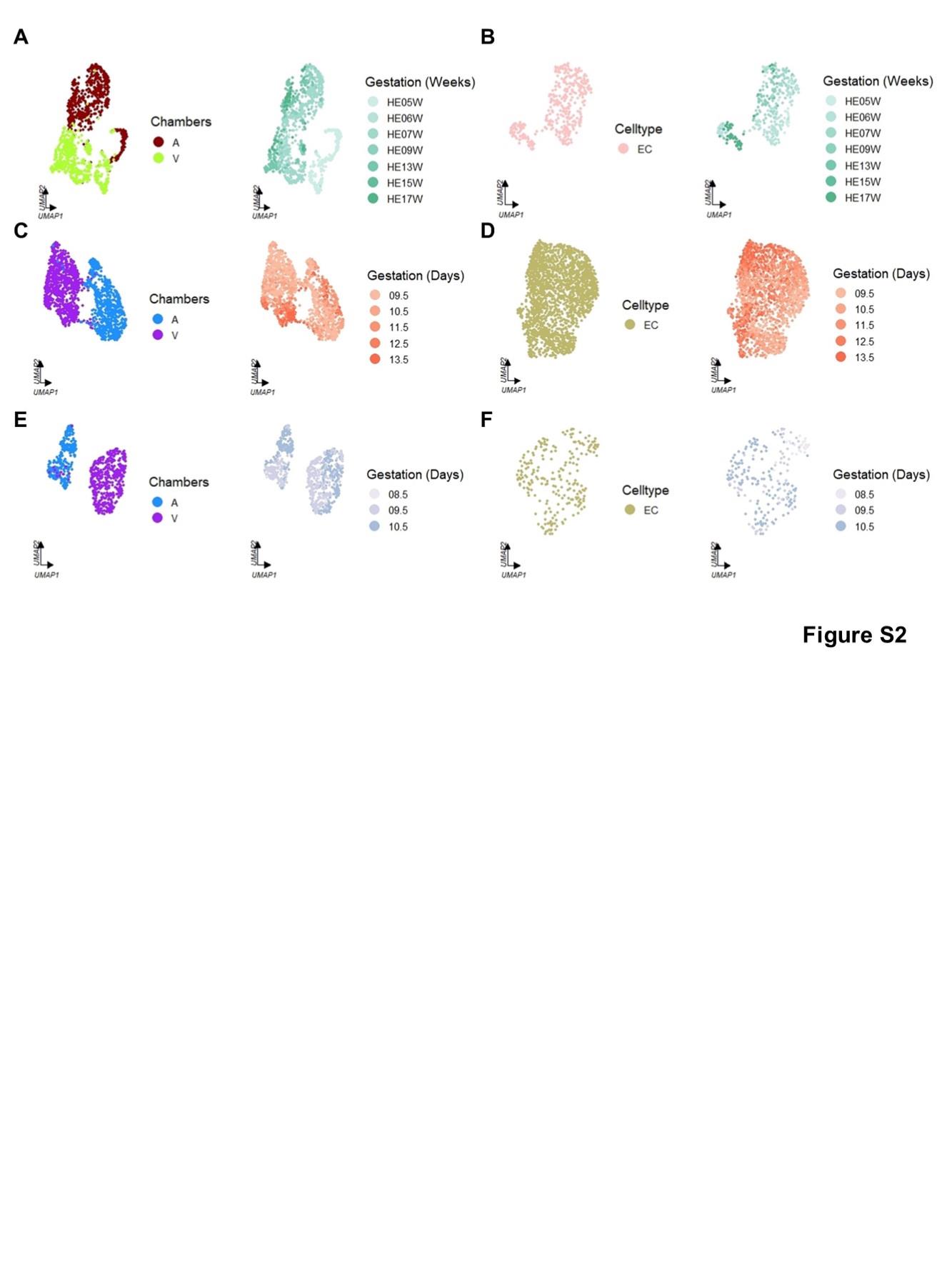

Supplement: Supplementary file 1 [file ijms-25-03240-s001.zip › Supplementary FIG 2.jpg]

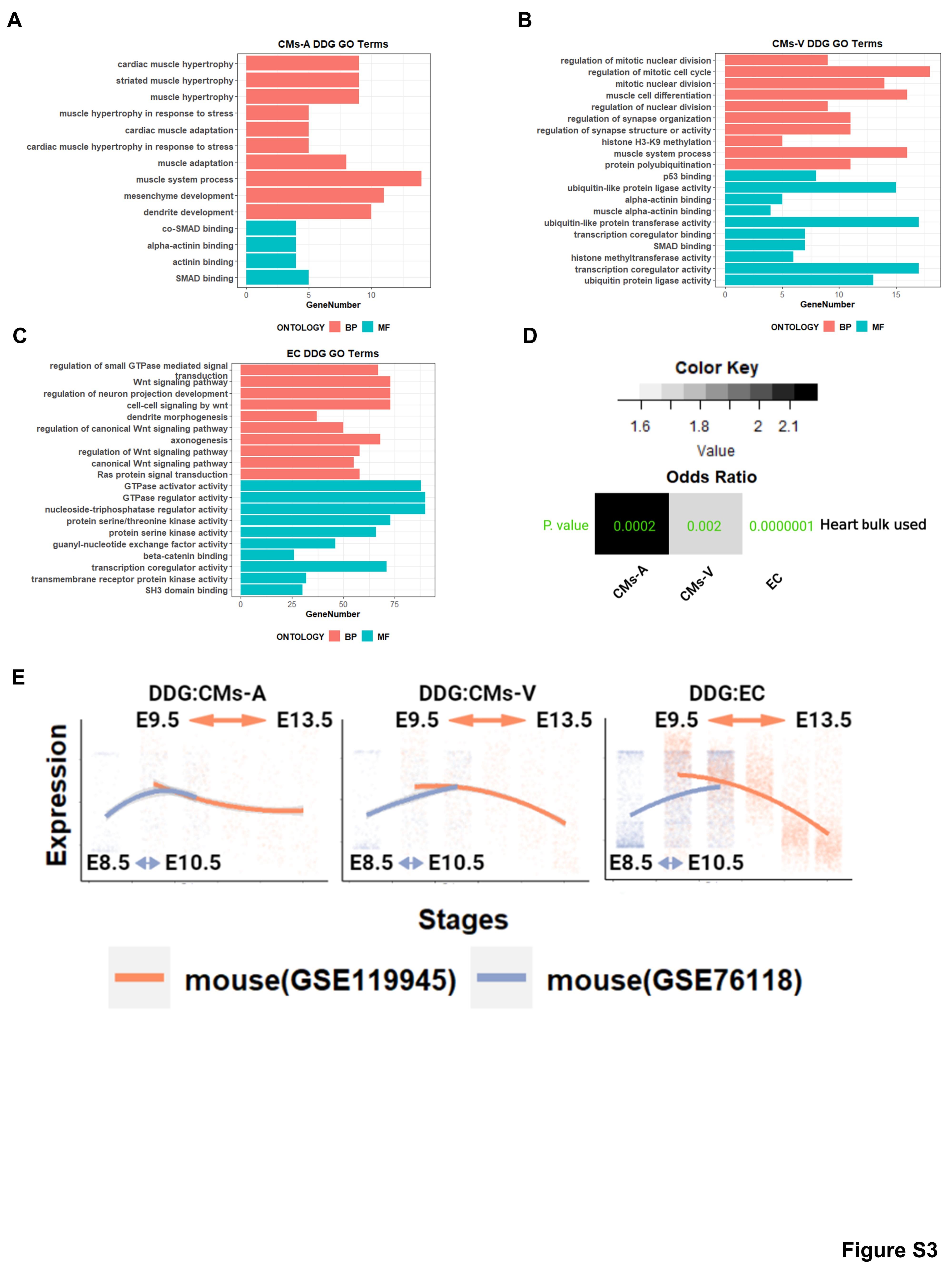

Supplement: Supplementary file 1 [file ijms-25-03240-s001.zip › Supplementary FIG 3.jpg]

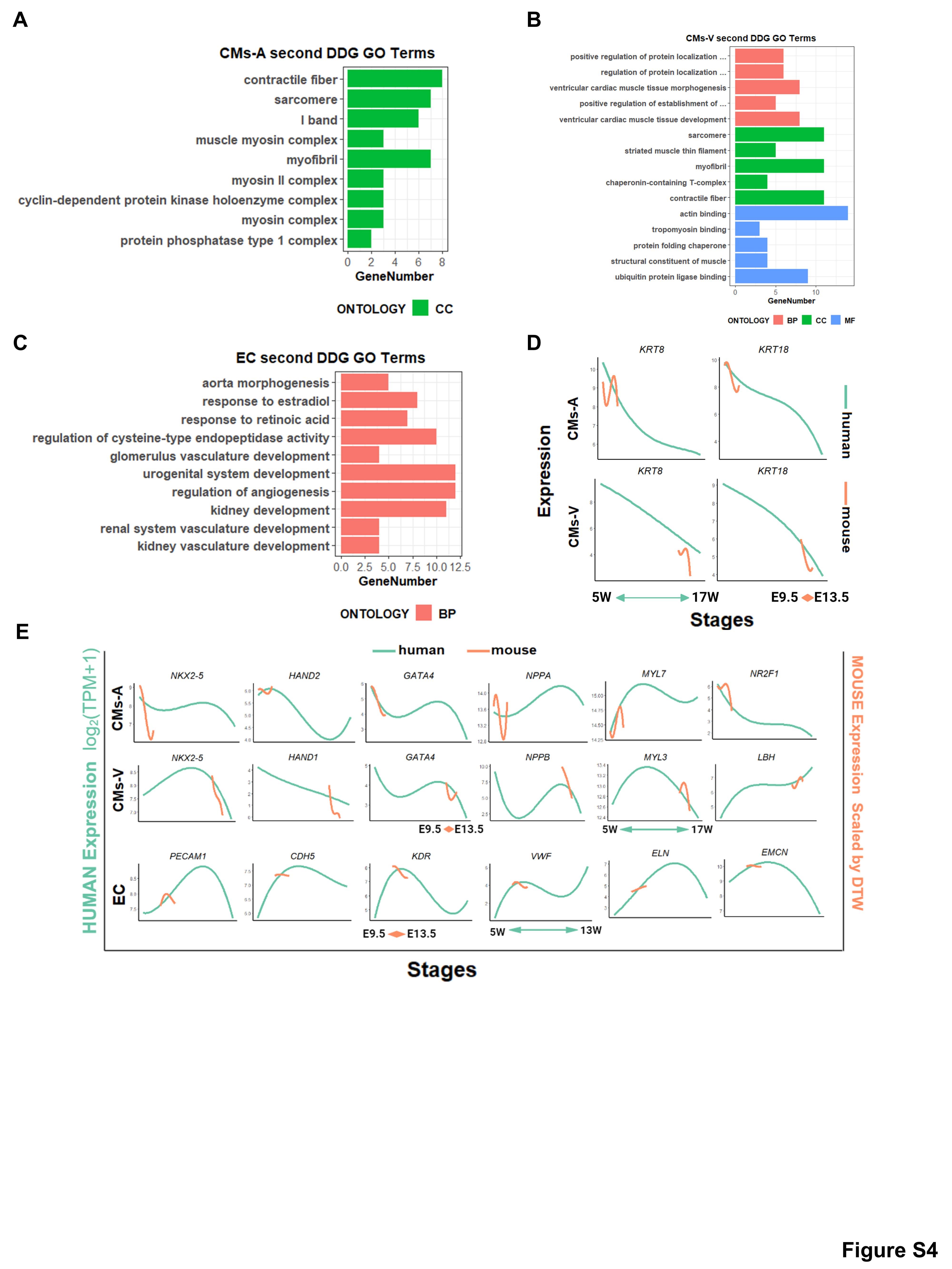

Supplement: Supplementary file 1 [file ijms-25-03240-s001.zip › Supplementary FIG 4.jpg]

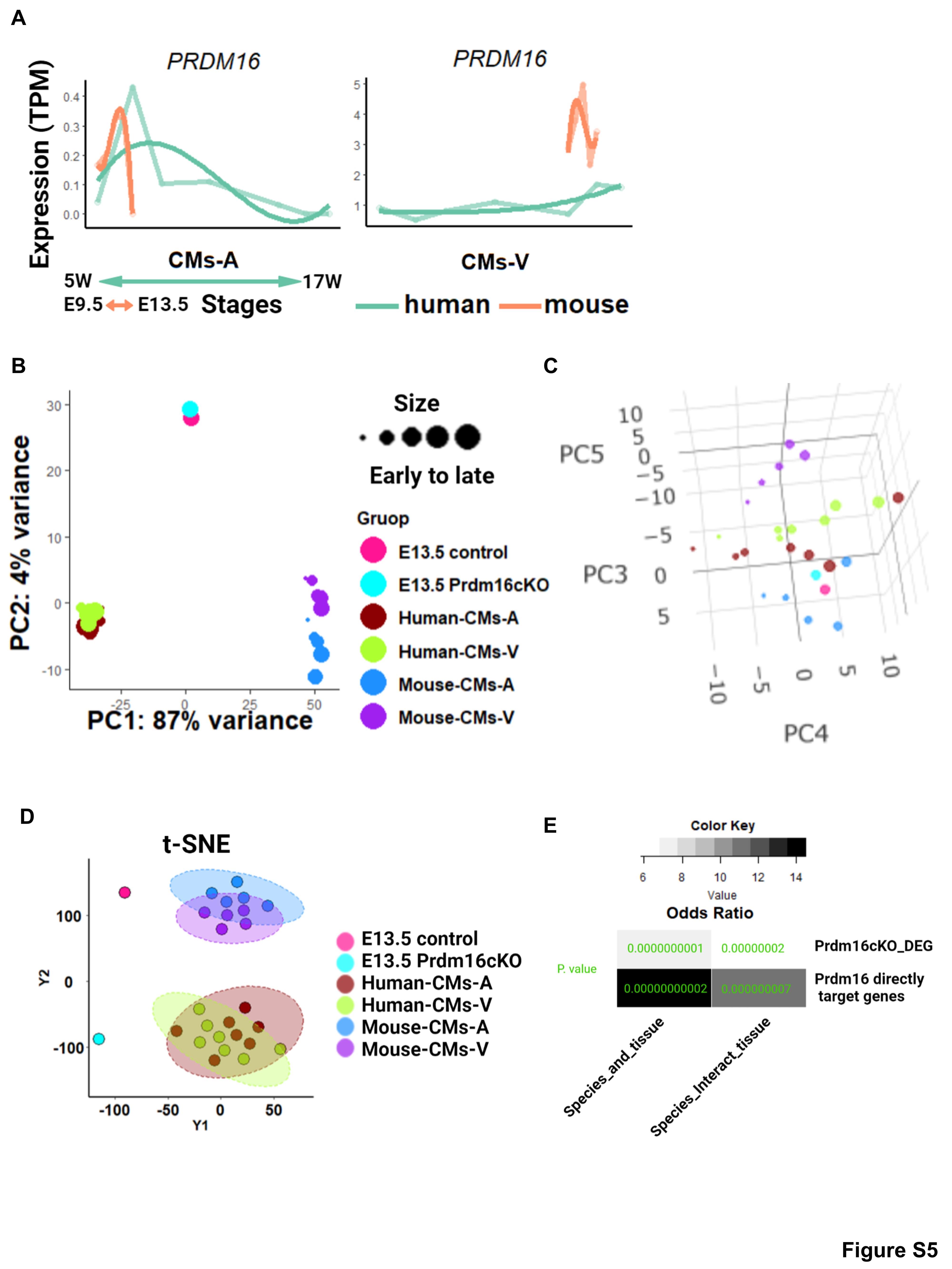

Supplement: Supplementary file 1 [file ijms-25-03240-s001.zip › Supplementary FIG 5.jpg]

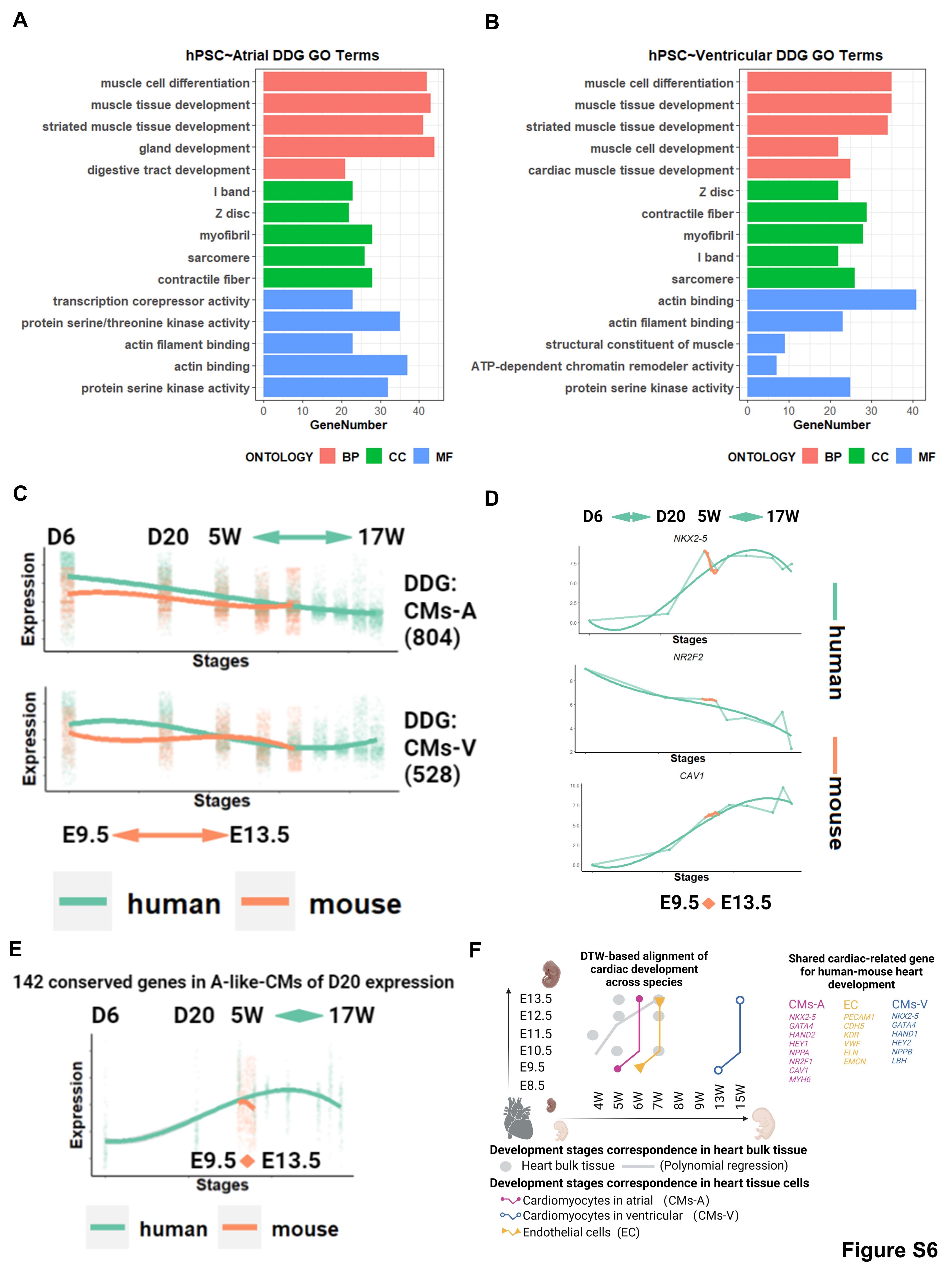

Supplement: Supplementary file 1 [file ijms-25-03240-s001.zip › Supplementary FIG 6.jpg]
